# Occurrence of *Echinococcus felidis* in Apex Predators and Warthogs in Tanzania: First Molecular Evidence of Leopards as a New, Definitive Host and Implications for Ecosystem Health

**DOI:** 10.3390/pathogens14050443

**Published:** 2025-04-30

**Authors:** Barakaeli Abdieli Ndossi, Eblate Ernest Mjingo, Mary Wokusima Zebedayo, Seongjun Choe, Hansol Park, Lee Dongmin, Keeseon S. Eom, Mohammed Mebarek Bia

**Affiliations:** 1 Tanzania Wildlife Research Institute, Arusha P.O. Box 661, Tanzania; barakan@gmail.com (B.A.N.); eblate.ernest@gmail.com (E.E.M.); mary.zebedayo@tawiri.or.tz (M.W.Z.); 2 International Parasite Resource Bank, Cheongju 28644, Republic of Korea; yes.hansol@gmail.com (H.P.); dongmin83@gmail.com (L.D.); 3 Department of Parasitology, Parasitology Research Center Chungbuk National University, School of Medicine, Cheongju 28644, Republic of Korea; vetazmo@gmail.com

**Keywords:** leopards, warthogs, lions, *Echinococcus felidis*, Serengeti ecosystem

## Abstract

(1) Background: Limited information on *Echinococcus* species among the wildlife in Tanzania has created a significant knowledge gap regarding their distribution, host range, and zoonotic potential. This study aimed to enhance the understanding of *Echinococcus felidis* transmission dynamics within the great Serengeti ecosystem. (2) Methods: A total of 37 adult *Echinococcus* specimens were collected from a leopard (*Panthera pardus*) (n = 1) in Maswa Game Reserve and 7 from a lion (*Panthera leo*) (n = 1) in Loliondo. Two hydatid cysts were also obtained from warthogs (n = 2) in the Serengeti National Park. (3) Results: Morphological examination revealed infertile cysts in warthogs that were molecularly identified as *E. felidis*. This marks the first molecular evidence of *E. felidis* in leopards and warthogs in Tanzania. Pairwise similarity analysis showed 98.7%–99.5% identity between Tanzanian, Ugandan, and South African isolates. Thirteen unique haplotypes were identified, with a haplotype diversity of (Hd = 0.9485) indicating genetic variability. Phylogenetic analysis grouped *E. felidis* into a single lineage, with the leopard isolate forming a distinct haplotype, suggesting leopards as an emerging host. Lion and warthog isolates shared multiple mutational steps, suggesting possible genetic divergence. (4) Conclusions: This study confirms African lions and leopards as definitive hosts and warthogs as potential intermediate hosts of *E. felidis* in the Serengeti ecosystem. Our findings highlight disease spillover risks and stress the importance of ecosystem-based conservation in wildlife–livestock overlap areas. Although *E. felidis* is believed to be confined to wildlife, the proximity of infected animals to pastoralist communities raises concerns for spillover. These findings highlight the importance of ecosystem-based surveillance, especially in wildlife–livestock–human interface areas.

## 1. Introduction

Echinococcosis is a major zoonotic disease caused by the larval (metacestodes) stage of *Echinococcus* (Cestoda: Taeniidae), imposing significant global medical and economic burdens [1,2]. The genus *Echinococcus* comprises several genetically distinct species, such as *E. granulosus* sensu lato (G1–G10), *E. multilocularis*, *E. vogeli*, *E. oligarthra*, and *E. shiquicus* [3,4]. Within the *E. granulosus* complex, molecular studies have revealed cryptic species with specific hosts associations and geographical distributions. These include *E. granulosus* sensu stricto (s. s.) (G1, G3), *E*. *equinus* (G4), *E*. *ortleppi* (G5), *E*. *canadensis* (G6–8, G10), and *E*. *felidis*, each exhibiting unique host specificity and epidemiological significance [5,6,7,8,9]. Adult *Echinococcus* spp. primarily infect definitive hosts such as domestic dogs (*Canis familiaris*) and wild canids [10,11,12,13]. Gravid proglottids are shed in feces, and the released eggs contaminate the environment, where they can be ingested by intermediate hosts, resulting in Cyst echinococcosis (CE) predominantly in the liver and lungs [14,15,16,17,18].

Humans serve as accidental intermediate hosts, becoming infected through ingestion of eggs via contaminated food, water, or fomites [17]. The disease can lead to severe clinical outcomes in both humans and animals, including organ failure, secondary infections, and death [19,20,21]. Despite its endemic status in several African countries, molecular clarification on *Echinococcus* spp., particularly in wildlife, remains limited. Existing studies are primarily from Mauritania [22], Sudan [23,24], Ethiopia [25,26], Somalia [27], Namibia [28], and Kenya [12,13,29,30,31,32], with limited references to Tanzania [33]. Reports of *E*. *felidis* in wildlife hosts have emerged from South Africa [34,35], Uganda [31,36], Kenya [37,38], and Namibia [39].

Lions (*Panthera leo*), spotted hyenas (*Crocuta crocuta*), and domestic dogs (*Canis familiaris*) have been identified as definitive hosts of *E. felidis*, whereas warthogs (*Phacochoerus africanus*) and hippopotamuses (*Hippopotamus amphibius*) are among the few documented intermediate hosts [33,35,40]. Molecular diagnostic tools targeting genes such as cytochrome oxidase subunit 1 (*Cox 1*), NADH dehydrogenase gene (*nad1*), ribosomal DNA (ITS1 & ITS2), and RAPD-PCR have enabled the precise identification and genotyping of *Echinococcus* species [27,37,41,42]. However, the epidemiology, geographic distribution, and host range of *E. felidis* in sub-Saharan Africa, especially Tanzania, remain poorly understood [43].

In Tanzania, Copro-ELISA has detected *Echinococcus* antigens in cheetahs, lions, and spotted hyenas [44]; however, this method may cross-react with other cestodes and lacks species level specificity [45,46]. Therefore, molecular studies are essential to better understand CE epidemiology and transmission dynamics within wildlife, livestock, and human interfaces [47]. Previous studies have emphasized the value of molecular characterization for clarifying taxonomy, epidemiology, host specificity, and ecology of *Echinococcus* spp., particularly in regions like sub-Saharan Africa that are underrepresented [48]. Additionally, an integrated One Health approach combining molecular and ecological studies is needed to assess zoonotic risks and inform control strategies [49]. Although Tanzania is recognized for its mega biodiversity in East Africa [50,51], molecular data on *Echinococcus* spp., including *E. felidis* in wildlife, remain scarce. This gap hinders a broad understanding of transmission patterns and epidemiological risks to livestock and humans. This study provides the first molecular confirmation of *E. felidis* in lions and leopards as definitive hosts and warthogs as a suspected intermediate host in the Serengeti ecosystem, providing insights into its transmission dynamics in a landscape shared by wildlife, livestock, and humans [52]. These findings provide baseline data that contribute to a better understanding of the presence of *E. felidis* in the Serengeti ecosystem, emphasizing the need for broader surveillance and targeted research to enhance ecosystem health and inform One Health-based control strategies.

## 2. Materials and Methods

### 2.1. Study Area

**Figure 1:**
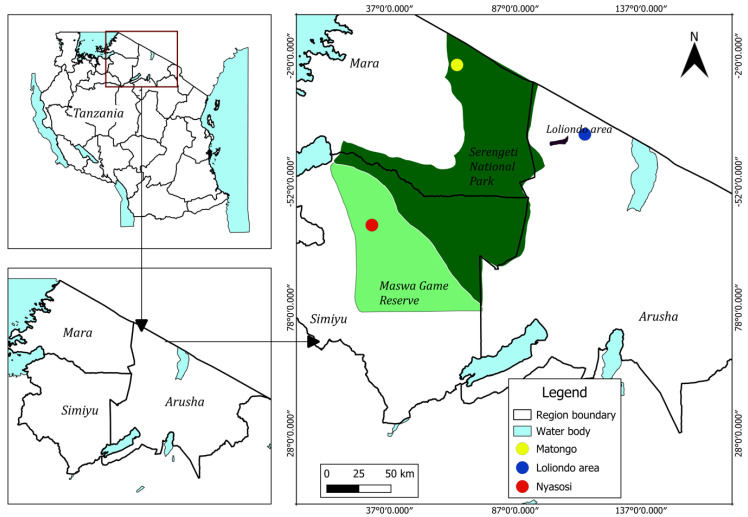
Study area within the Serengeti–Mara ecosystem.

This study was conducted between 2014 and 2024 in Serengeti National Park, Maswa Game Reserve, and the Loliondo area within the greater Serengeti ecosystem in Tanzania (Figure 1). A total of 37 adult *Echinococcus* spp. specimens were collected from the small intestine of a leopard (*Panthera pardus*) (n = 1) in the Maswa Game Reserve (S 3°18′43.07400″ S″, 34°30′30.85560″ E″), and 7 specimens were recovered from the small intestine of a lion (*Panthera leo*) (n = 1) in the Loliondo area (2°22′52.65120″ S″, 35°16′51.16440″ E″). Two hydatid cysts were collected from the lungs of the two warthogs (*Phacochoerus africanus*) (n = 2) along the Matongo area (1°38′35.81650″ S″, 34°6′13.50080″ E″) in Serengeti National Park. The cysts were spherical, thin-walled, and measured approximately 2–3 cm in diameter. Morphological examination revealed that the cysts were infertile, as no protoscoleces were observed. All animals were found dead during routine wildlife disease surveillance within the Serengeti ecosystem. Due to the conservation status of these species in Tanzania, sampling was opportunistic and restricted to carcasses obtained through authorized surveillance activities.

Each intestine was sectioned and opened lengthwise, and the contents were washed with physiological saline (0.85%) to isolate the parasites under a stereoscopic microscope [53]. Adult worms were preserved in 10% formalin for morphological examination and in 70% ethanol for molecular studies.

The collected cysts were prepared according to Thompson and McManus’ method [54]. Briefly, the hydatid liquid was drained from the collected cysts by using a 0.5 mm sterile syringe and stored in 15 mL sterilized and labelled falcon tubes with the immediate addition of 70% ethanol, then kept at room temperature. In addition, the germinal membranes from the cysts were collected, and Thompson’s procedures were utilized until further use [54].

### 2.2. Morphological Analysis

For the morphological analysis, a subset of 15 adult worms (10 from the leopard and 5 from the lion) were stained by using Semichon’s acetocarmine. Prior to staining, the worms were cleaned in a physiological saline solution (0.85%) to remove debris and mucus from the tegument. After cleaning, the worms were transferred to 70% ethanol for 30 min and transferred to Semichon’s acetocarmine stain for 10 h. The worms were destained in a solution of acid ethanol (Ethanol 70% with 1 mL 1 N HCl/10 mL), followed by dehydrating in 70% ethanol for 30 min, 80% Ethanol for 30 min, 90% ethanol for 30 min, and 100% × 2 ethanol for 30 min. In the last step, the worms were cleaned in xylene for 1 min and mounted on the slide by Canada balsam [55]. Morphometric parameters such as total body length, the number and size of proglottids, scolex structure, and rostellar hooks were measured and compared. Although 7 adult worms were recovered from the lion, the poor preservation state of these samples post-collection prevented detailed morphological study. Only molecular identification was performed on these specimens.

### 2.3. DNA Extraction and PCR Amplification

DNA was extracted from 7 individual adult worms (5 from the leopard and 2 from the lion) and from each warthog cyst using the DNeasy Blood and Tissue Kit (QIAGEN^®^, Hilden, Germany) according to the manufacturer’s protocol with some modifications. For the larval stage, small (<2 mm) pieces of cysts, previously kept at 4 °C in 70% ethanol, were prepared and washed with PBS x1 using a shaker (LABOGENE R100) overnight to thoroughly centrifuge the membrane at (15,345× *g*). Protoscoleces and adult worms were washed with PBS X1, centrifuged at (15,345× *g*), and had the supernatants discarded a total of three times. Immediately, the samples were crushed with a sterile pestle, and genomic DNA was extracted and kept at −20 °C until further use.

The polymerase chain reaction (PCR) was carried out targeting the Cytochrome C oxidase 1 (*Cox 1*) from mitochondrial genomes. The primers JB3 (5′-TTT TTT GGG CAT CCT GAG GTT TAT-3′) and JB4.5 (5′TAA AGA AAG AAC ATA ATG AAA ATG-3′) were used for the amplification of the *Cox 1* gene region [56]. The PCR cocktail was prepared by using 8 μL HiPi Plus 5× PCR Master Mix, 12.5 pmol forward primer, 12.5 pmol reverse primer, 26 μL distilled water, and 4–20 ng DNA sample to make a final volume of 40 μL. The reaction was carried out in an automatic thermal cycler whereby the pre-denaturation was set for 3 min at 95 °C. Thirty-five cycles were well-set whereby in each cycle, DNA separation was conducted for 30 s at 95 °C, annealing for 30 s at 47 °C, and the extended time for DNA synthesis was 1 min for 72 °C, with a final extension step at 72 °C for 10 min. The PCR products were run on 1% agarose gel, and the images were obtained on a gel-imaging device (Gel Doc XR + System).

### 2.4. Molecular Analysis

The sequencing was conducted in a biomolecular company (Cosmogenetech Co., Ltd., Seoul, Republic of Korea). The sequence analysis and alignment of sequences were performed by Geneious software Version 9.0. [57]. The sequenced samples were trimmed and assembled using de novo sequence assemblers to generate the consensus sequences. As a result, *Cox1* sequences of 228 bp from the lion, 258 bp from the leopard, and 224 bp from the warthog were obtained. The National Center for Biotechnology Information (NCBI) https://blast.ncbi.nlm.nih.gov/Blast.cgi (accessed on 10 March 2025) was used to determine sequence similarity and identity matches with existing reference sequences.

The obtained sequences were compared with the sequence of *E. felidis* and with other *Echinococcus* species from the GenBank (Table 1) to observe the phylogenetic relationships between individuals. In addition, *Taenia solium* was added in the analysis as an outgroup [58]. Using MEGA v.6. software [59] and the Maximum Likelihood method [60] to root the phylogenetic tree, Bayesian Information Criterion (BIC) value was determined with the HKY + I (Hasegawa–Kishino–Yano + invariant sites) method, with 1000 bootstrap replication estimated to obtain a high level of confidence. The haplotype networks were generated according to mutation steps by using the Network software (PopArt V.1.7) that depends on statistical parsimony after creating the data file for the *Cox 1* genetic locus by utilizing DnaSP v.6. software [61].

## 3. Results

### 3.1. Morphological Identification of Echinococcus felidis

**Figure 2:**
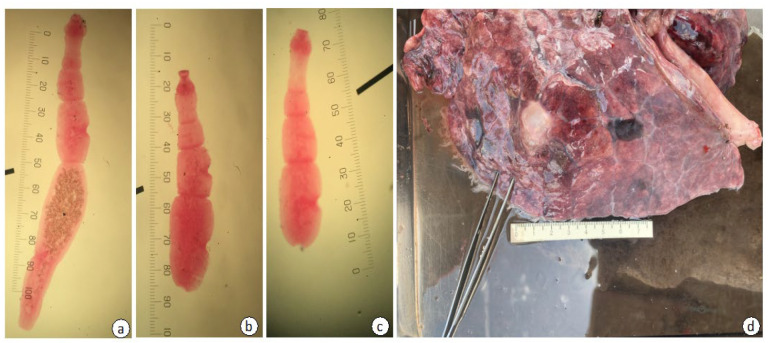
Morphological examination of adult *E. felidis* from the leopard (**a**–**c**) showing strobila composed of 4–5 segments, with a total length ranging from 1.65 to 3.10 mm (av.2.02 mm). A hydatid cyst (**d**) in the lung of a warthog measured approximately 2 × 3 cm in diameter.

The morphometric features of *E. felidis* worms from the leopard were as follows: having four to five proglottids with a total body length ranging from 1.65 to 3.10 mm (average 2.02 mm) (Figure 2). For instance, the gravid proglottids located at the posterior end of the strobila contained immature eggs within the uterus, measuring approximately 29.63 × 39.50 µm (Figure 2). The genital pore was positioned near the middle to posterior region of the proglottids, while the number of testes varied between 12 and 60, and they were distributed both anterior and posterior to the genital pore. The uterine structure was sac-like, with no lateral branches (Figure 2), while the cirrus sac, measuring between 69.13 and 188.89 µm in length, was slightly smaller compared to other species (Table 2).

### 3.2. Molecular Identification of Echinococcus felidis and Phylogenetic Analysis

**Figure 3:**
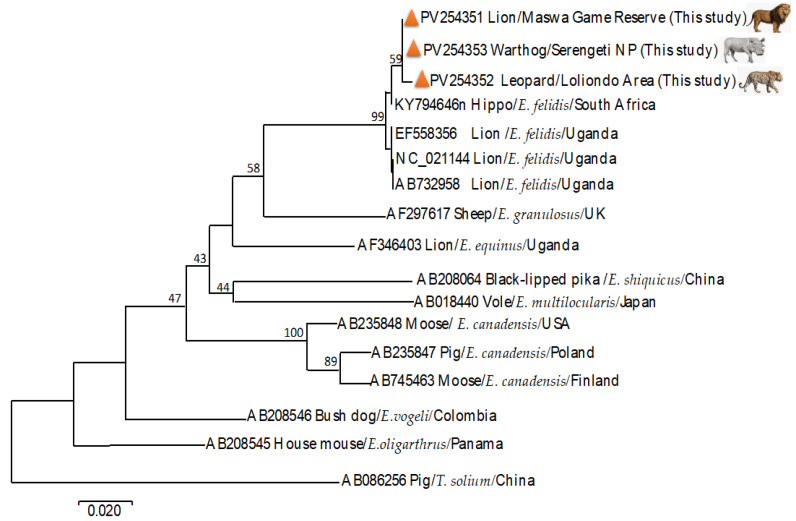
Phylogenetic relationship tree of the Mitochondrial *Cox* 1 gene of adult worms and hydatid cysts from the African lion, leopard, and warthog rooted with different sequences of *Echinococcus* spp. The tree shows the samples from Tanzania grouped with *Echinococcus felidis* sequences already published.

The obtained sequences were aligned with the published *Echinococcus* sequences from GenBank (Table 1). Pairwise similarity analysis revealed intraspecies variation (98.7%–99.5%) among *E. felidis.* The leopard isolate (PV254352) exhibited 98.7%–99.5% similarity with sequences NC_021144, AB732958, EF558356, and KY794646, while the lion isolate (PV254351) showed 99.7%–99.8% similarity with the same sequences (Figure 3). *E. felidis* from the warthog (PV254351) showed high genetic similarity with both lion- and leopard-derived *E. felidis* sequences. The close genetic relationship revealed minimal divergence among hosts. For instance, the warthog sequence showed 99.6% similarity with the lion (PV254351) and 99.5% similarity with the leopard (PV254352) sequences. Additionally, *E. felidis* from the warthog exhibited a high similarity of 99.7% to 99.8% with sequences from other regions, including NC_021144, AB732958, EF558356, and KY794646.

The maximum likelihood analysis revealed the phylogenetic clusters among the sequences with a high level of confidence (>95%). It placed the *E. felidis* sequences into a single cluster (Figure 3). The obtained genetic distance of 0.12 to 0.18 showed that *E. felidis* forms a distinct lineage compared with other *Echinococcus* species (Figure 3).

**Figure 4:**
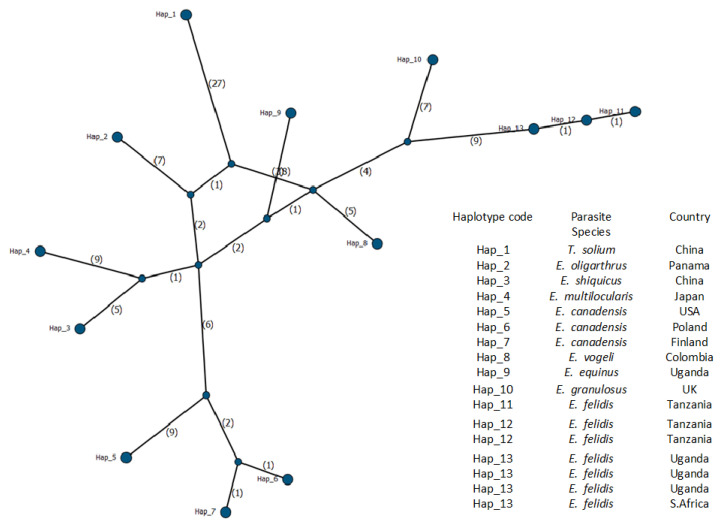
Median-joining haplotype network of 13 mitochondrial *Cox*1 haplotypes of *Echinococcus* spp. Haplotypes are represented by circles scaled to sequence frequency. *E. felidis* forms a distinct cluster (Hap_11–Hap_13), with Hap_13 linking previously reported isolates from Uganda and South Africa. Hap_12 includes lion and warthog samples from this study, while Hap_11, from a leopard, appears genetically distinct. The central position of Hap_8 suggests an ancestor haplotype, with peripheral haplotypes (Hap_1–Hap_7) reflecting host and geographic divergence.

Genetic diversity and haplotype distribution were generated among the *E. felidis* and other *Echinococcus* species from various regions. A total of 17 sequences (Table 1) were used for the analysis. Only 189 informative sites were considered after excluding missing data and sites with gaps to identify genetic variation among the sequences. Among these, 57 variable sites were identified, reflecting genetic polymorphism. Among the haplotypes identified, Hap_13 was shared across different hosts, such as African lion and hippopotamus isolates from Uganda and South Africa, suggesting a widespread and possibly conserved lineage; the overall haplotype diversity (Hd) was 0.9485, indicating high genetic variation within the dataset (Figure 4).

The haplotype network revealed 13 unique haplotypes, 11 of which were represented by single sequences (Figure 4). *E. felidis* formed a distinct lineage clustered on a single branch that includes Hap_11, Hap_12, and Hap_13. Among these, Hap_13 appears to be the central haplotype, linking *E. felidis* with closely related species. Hap_12 comprises two sequences identified in the present study, one originating from a lion and the other from a warthog. The cysts recovered from the warthog were morphologically characterized as infertile. Genetic analysis confirmed the presence of *E. felidis.* Despite this finding, the role of warthogs as an intermediate host for *E. felidis* remains undefined. Hap_13 includes four previously reported *E. felidis* from Uganda (NC_021144, AB732958, EF558356) and South Africa (KY794646). The isolate from the leopard in this study (PV254352) formed Hap_11, which appeared genetically distinct and potentially more recently derived. Its position in the network suggests a unique lineage or emerging variant, supporting the possibility of the leopard as a new or incidental host for *E. felidis*.

Additionally, several haplotypes radiated from the central Hap_8, which may represent the common ancestor for the broader *Echinococcus* spp. lineage in this study. In contrast, Hap_1 through Hap_7 formed separate branches, reflecting host-specific divergence, geographic isolation, or localized evolutionary adaptations (Figure 4).

## 4. Discussion

Infection with *Echinococcus* spp. has been reported in various wildlife species across several African countries [69]. African lions, spotted hyenas, jackals, and wild dogs are among the definitive hosts reported with *Echinococcus* spp. infections [37]. Common intermediate hosts susceptible to CE include a wide range of wild herbivores, such as hippopotamuses, waterbucks, buffalo, warthogs, blue duikers, wildebeests, impalas, topis, baboons, giraffes, gazelles, and zebras [40]. These species serve as natural reservoirs for larval stages of *Echinococcus* spp. that are essential for maintaining the sylvatic cycle [40]. Although the hydatid cysts recovered from the warthog were infertile, molecular identification in this study provides preliminary evidence confirming *E. felidis*, suggesting that warthogs can harbor this species, yet its role as an intermediate host remains inconclusive.

The spatial distribution of *Echinococcus* spp. infections in wildlife species is influenced by ecological factors, landscape features, and regional climate, all of which affect the survival and viability of *Echinococcus* spp. eggs in the environment [70,71]. This study provides the first molecular confirmation of *E. felidis* in Tanzania, identifying an African lion and a leopard as definitive hosts. The detection of *E. felidis* in the leopard represents a new host record, raising important questions about host adaptability, predator–prey dynamics, and potential cross-species transmission.

Morphometric features observed in this study align with previous descriptions of *E. felidis* [66,67,68]. However, morphology alone is insufficient for species confirmation due to variations caused by geographical differences, host adaptation, or intraspecific morphological plasticity [72]. The molecular identification of *Echinococcus* spp. in livestock and humans in Tanzania have been studied [47,73]. However, there is limited information about *Echinococcus* spp. infections in wildlife, with only Copro-ELISA studies identifying infections in spotted hyenas and cheetahs [44]. This study fills a critical knowledge gap by providing molecular evidence of *E. felidis* in wild carnivores and warthogs from the Serengeti ecosystem.

The observed genetic variation in *E. felidis* indicates species-specific divergence and gene conservation within the genus *Echinococcus* [74,75]. Although we observed divergence among the isolates from the lion, leopard, and two warthogs, the relatively short fragment of the *Cox*1 gene used in this study may limit the resolution of genetic differentiation [76]. While *Cox*1 is suitable for species-level identification, more robust phylogenetic analyses would benefit from longer mitochondrial fragments or whole-genome sequencing. Further research is necessary to clarify population structure, genetic variation, and host-specific adaptations [77,78]. Phylogenetic analysis using the maximum likelihood method demonstrated clear clustering of *E. felidis* sequences with strong bootstrap support (>95%). The phylogenetic tree also confirmed *E. felidis* as a sister taxon to *E. granulosus* (AF297617), consistent with previous findings [65].

Our findings are supported by dietary studies showing that apex predators are more exposed to *Echinococcus* spp. infection due to prey consumption pattens [79,80,81]. Host population density, availability of prey, reproduction, and habitat use predator–prey interactions and influence transmission dynamics [82,83]. The detection of *E. felidis* in a leopard may suggest either accidental infection or potential adaptability to other carnivores driven by overlapping ecological niches and/or environmental factors.

The proximity of the Maswa and Loliondo areas to pastoralist communities, particularly the Maasai and Sukuma tribes, increases the potential for human and livestock exposure to *Echinococcus* species, including *E. felidis* [84,85]. Human activities such as bushmeat hunting, livestock grazing, and encroachment into protected areas could facilitate cross-species transmission [86]. Although *E. felidis* has traditionally been thought to remain confined to wildlife [43], its potential for zoonotic transmission is a growing concern, especially with the expanding human–wildlife interface. The detection of *E. felidis* in multiple hosts coupled with observed genetic divergence suggests a dynamic sylvatic cycle with implications for ecosystem health.

Among the limitations of this study is the small sample size, comprising one leopard, one lion, and two warthogs collected opportunistically during surveillance activities. These species are highly protected and can only be sampled under special conditions or permits in Tanzania Protected Areas. Despite this limitation, our findings provide valuable insight into the ecology and transmission of *E. felidis.* Broader sampling is essential to fully understand the role of different hosts and the extent of transmission.

Our study emphasizes the need for considering interspecies parasite transmission in the management of carnivore and ungulate populations in protected areas. The close genetic relationship among *E*. *felidis* isolates from different host species highlights the risk of disease spillover. Conservation strategies must incorporate disease ecology, especially in areas where wildlife, livestock, and human populations intersect.

## 5. Conclusions

This study provides the first molecular confirmation of *Echinococcus felidis* in Tanzania, identifying an African lion and a leopard as definitive hosts within the Serengeti ecosystem. Although the cysts recovered from a warthog were infertile, molecular analysis confirmed the presence of *E. felidis* DNA. However, the absence of viable protoscoleces made its role in transmission inconclusive. While the warthog cannot be classified as an intermediate host, its contribution to the parasite’s life cycle should not be entirely excluded. Further research is needed to clarify its ecological role and to better understand the broader transmission dynamics of *E. felidis* in East Africa.

## Figures and Tables

**Table 1:** Reference sequences of *Echinococcus* species analyzed in this study.

Host Name	Scientific Name	Country	Parasite Species	Haplotype Code	Accession Number	References
Pig	*Sus scrofa*	China	*Taenia solium*	Hap_1	AB086256	[62]
House mouse	*Mus musculus*	Panama	*E. oligarthrus*	Hap_2	AB208545	[63]
Black-lipped pika	*Ochotona curzoniae*	China	*E. shiquicus*	Hap_3	AB208064	[63]
Vole	*Craseomys rufocanus*	Japan	*E. multilocularis*	Hap_4	AB018440	[64]
Moose	*Alces alces*	USA	*E. canadensis*	Hap_5	AB235848	[63]
Pig	*Sus scrofa*	Poland	*E. canadensis*	Hap_6	AB235847	[63]
Moose	*Alces alces*	Finland	*E. canadensis*	Hap_7	AB745463	[7]
Bush dog	*Speothos venaticus*	Colombia	*E. vogeli*	Hap_8	AB208546	[63]
African lion	*Panthera leo*	Uganda	*E. equinus*	Hap_9	AF346403	[65]
Sheep	*Ovis aries*	UK	*E. granulosus*	Hap_10	AF297617	[65]
African lion	*Panthera leo*	Tanzania	*E. felidis*	Hap_11	PV254351	This study
Leopard	*Panthera pardus*	Tanzania	*E. felidis*	Hap_12	PV254352	This study
Warthog	*Phacochoerus africanus*	Tanzania	*E. felidis*	Hap_12	PV254353	This study
African lion	*Panthera leo*	Uganda	*E. felidis*	Hap_13	NC_021144	[7]
African lion	*Panthera leo*	Uganda	*E. felidis*	Hap_13	AB732958	[7]
African lion	*Panthera leo*	Uganda	*E. felidis*	Hap_13	EF558356	[36]
Hippopotamus	*Hippopotamus amphibius*	S. Africa	*E. felidis*	Hap_13	KY794646	[34]

**Table 2:** Morphometric features of *E. felidis* isolates from the leopard in relation to others.

Feature	*E. felidis* from Leopard (This Study)	*E. felidis* Verster, 1965 [66]	*E. felidis* Ortlepp, 1935 [67]	*E. felidis* Raush, 1953 [68]
Body length (mm)	1.65–3.10 (av.2.02)	2.12–5.220 (av.3.239) ± 0.3	3.42–5.22 (av.4.21 ± 0.6)	6 mm
Number of segments	4.0–5.0	3 (18.5%), 4 (47.0%), 5 (34.5%)	4–5 usually 4	3.0–4.0
Number of hooklets	28–32	-	-	-
Number of testes	12.0–60.0	28–45 (av.35.9 ± 3.5)	30–46 (av.6 ± 4.2)	32–46
Cirrus sac	69.13–188.89 (av.128.39 µm long, 49.38–164.19 µm (av.88.88 µm))	Mt: 82.8–202.4 (129.4 ± 2.4) µm long, 46.0–105.8 (64.9 ± 99) µm with gravid segment 147.2–184 (159.9 ± 13.7 µm, 56.0–79.2 (717 ± 9.3))	Mt: 115.0–174.8 (148.2 ± 15.5 µm long, 55.2–92.0 (77.3 + 133)) width. Gravid segment 142.6–207.0 (176.9 ± 21.6) µm, 64.4–96.6 (83.2 ± 10.2) µm	-
Egg size	29.63 × 30.50 µm	-	-	37–40.5 (32–35.5 µm)

## Data Availability

All Raw-DNA sequences were deposited in The National Center for Biotechnology Information (NCBI) PV254351-PV254353.
